# Ethical Considerations for Treating Cancer Patients During the SARS-CoV-2 Virus Crisis: To Treat or Not to Treat? A Literature Review and Perspective From a Cancer Center in Low-Middle Income Country

**DOI:** 10.3389/fmed.2020.561168

**Published:** 2020-10-07

**Authors:** Amal Al-Tabba', Maysa Al-Hussaini, Razan Mansour, Hala Sultan, Hikmat Abdel-Razeq, Asem Mansour

**Affiliations:** ^1^Independent Researcher, Amman, Jordan; ^2^Department of Pathology and Laboratory Medicine, King Hussein Cancer Center, Amman, Jordan; ^3^Human Research Participants Protection Office, King Hussein Cancer Center, Amman, Jordan; ^4^Office of Scientific Affairs and Research, King Hussein Cancer Center, Amman, Jordan; ^5^University of Jordan School of Medicine, Amman, Jordan; ^6^Department of Medical Oncology, King Hussein Cancer Center, Amman, Jordan; ^7^King Hussein Cancer Center, Amman, Jordan

**Keywords:** pandemic, ethics, cancer care, guidelines, SARS-CoV-2, COVID-19

## Abstract

Providing routine healthcare to patients with serious health illnesses represents a challenge to healthcare providers amid the SARS-CoV-2 pandemic. Treating cancer patients during this pandemic is even more complex due to their heightened vulnerability, as both cancer and cancer treatment weaken the immune system leading to a higher risk of both infections and severe complications. In addition to the need to protect cancer patients from unnecessary exposure to SARS-CoV-2 infection during their routine care, interruption, and discontinuation of cancer treatment can result in negative consequences on patients' health, in addition to the ghost of rationing healthcare resources in high demand during a global health crisis. This article aims to explore the ethical dilemmas faced by decision-makers and healthcare providers caring for cancer patients during the SARS-CoV-2 pandemic. This includes setting triage criteria for non-infected cancer patients, fairly allocating limited healthcare resources between cancer patients and SARS-CoV-2 patients, prioritizing SARS-CoV-2 treatment or vaccine, once developed, for cancer patients and non-cancer patients, patient-physician communication on matters such as end-of-life and do-not-resuscitate (DNR), and lastly, shifting physicians' priorities from treating their own cancer patients to treating critically ill SARS-CoV-2 infected patients. Ultimately, no straightforward decision can be easily made at such exceptionally difficult times. Applying different ethical principles can result in very different scenarios and consequences. In the end, we will briefly share the experience of the King Hussein Cancer Center (KHCC), the only standalone comprehensive cancer center in the region.

## Introduction

In December 2019, the World Health Organization (WHO) in China was informed of cases of pneumonia of an unknown cause detected in Wuhan City, Hubei Province, now known as the novel coronavirus or SARS-CoV-2 (1). As of 07th August 2020, the virus had made its way to 188 countries causing a pandemic with almost 20 million confirmed cases (2). This rapid spread of the virus around the world spared healthcare providers and healthcare systems very little time, resulting in multiple medical and ethical mysteries, they are still struggling to unravel. Cancer care amidst the pandemic is in itself a mystery; as patients with cancer carry a higher risk of SARS-CoV-2 infection, Intensive Care Unit (ICU) admissions or even death compared with other patients (3). For example, after applying universal microbiologic screening for asymptomatic cancer patients in one hospital in the United Arab Emirates (UAE), 8.24% (7 out of 85) of the tested patients were positive for SARS-CoV-2 (4).

“What a terrible time to have cancer,” read the headline of an article at The Guardian written by Heather Chaney in her weekly column, describing difficulties in the treatment journey in the middle of the SARS-CoV-2 pandemic (5). Cancer patients and their families experience substantial concern and fear of this virus.

Just like cancer and its treatment may decrease the patient's ability to fight the infection, protective measures against the virus and limitation of health resources may also cause a delay in cancer treatment too. Thus, many ethical questions arise including how to sort cancer patients into prioritized and agreed-on categories? Who is to be treated first, patients with urgent medical needs, or those with the best chances of survival? Are there specific guidelines for cancer care during crisis and shortage of supplies? And what specific guidelines are there for healthcare providers treating cancer patients?

## Literature Review

Thereupon, some strategies and guidelines were proposed for cancer patients amid the SARS-CoV-2 crisis (6, 7). Triaging patients was of the most concerning challenge as identification of symptomatic patients with a suspicion of infection is crucial for the protection of other patients and healthcare providers. Screening points were allocated to entry sites of some cancer centers for patients, visitors, and even healthcare providers. Limitation of the number of visitors and providers was also recommended, and early-detection screening appointments were deferred. Regarding outpatient clinic visits, many were rescheduled or substituted with telemedicine when possible.

One controversial strategy was the intentional postponing of adjuvant chemotherapy, radiotherapy, stem cell transplant procedures and elective surgeries, which raises a question on how to balance a delay in cancer management against the risk of infection with SARS-CoV-2. This becomes even more baffling in certain aspects of cancer treatment. For example, hematologic malignancies require prompt diagnosis and treatment, whereas, most solid cancers may have longer treatment windows. Is it ethical to delay treatment in older patients and patients with metastatic disease where time is critical and delay may lead to worsening status and loss of the opportunity to treat? Additionally, should these decisions be unilateral, even when patients and physicians do not meet face-to-face?

Some cancer patients, in particular, are at a higher risk of becoming seriously ill if infected with SARS-CoV-2, these include patients receiving immunosuppressive therapy, targeted cancer treatments, recent bone marrow or stem cell transplants, or who are still taking immunosuppressive drugs in addition to patients with hematological malignancies (8). Revisiting the treatment plan for such patients is advisable. Patients and their healthcare providers should discuss whether the risks of beginning or continuing their cancer treatment could outweigh the benefits (8).

Moreover, as the number of SARS-CoV-2 cases is exponentially increasing, hospitals and cancer centers should expect a surge of cases into their wards, depleting its beds, equipment, and resources. Healthcare providers and patients will be faced with difficult choices. Therefore, setting an ethical triage criterion for non-infected cancer patients is of utmost importance.

## Triaging

Several triage strategies can be followed, each is based on a different ethical justification. Table 1 lists the different triaging strategies that could be followed as per the WHO (9).

**Table 1:** Different triage strategies (9).

**Triage criteria**	**Ethical justification**
1-Save the greatest number of people	This criterion directs us to give priority in allocation decisions to the category or categories of people that will result in the most lives saved. This usually involves allocating resources on the basis of a patient's prognosis and the amount of resources and/or personnel that will be required to sustain life.
2-First come, first served	This criterion directs us to give priority in allocation decisions to whoever accesses the resource first, independent of the severity of medical need or the needs of others. This criterion is based on the assumption that everyone has an equal ability to access the relevant resource—a presumption that is questionable during an emergency.
3-Protect the most vulnerable	This criterion directs us to give priority in allocation decisions to the most vulnerable category or categories of people in an emergency. Depending on the nature of the emergency, the most vulnerable groups could include infants, elderly people, pregnant women or people with particular medical conditions (e.g., obesity). If this criterion is chosen, we should give priority for live-saving interventions to members of vulnerable groups.
4-Equal access	This criterion directs us to give everyone (or at least similar categories of people) equal access to the benefit(s) of a resource when it is distributed, or at least an equal chance of accessing the benefits. If this criterion is chosen, no person should be given priority over another: each person is as important as any other, and all have an equal claim to access the resource. This differs from the “first come, first served” criterion in that its aim is to provide equal access to as many people as possible, not just those who access it first. Another version of this criterion is that if equal access cannot be given, an equal chance to access the benefits should be given; for instance, through a lottery process in which people who will receive a resource are chosen randomly.
5-Priority for the most important	This criterion directs us to allocate resources in such a way as to ensure that the individuals who are most important for society are given priority for access. The importance of individuals is usually understood in terms of who contributes most to the stability and protection of society (e.g., first responders, health care workers). If this criterion is chosen, individuals judged as having such a social function are given priority over those who do not.

Applying different triaging strategies to the same population (i.e., non-infected cancer patients) will give very different results all of which can be considered ethically justifiable. Health status and comorbidities, site and stage of cancer, and type of treatment and prognosis, all have to be weighed against the ethical principle adopted. For example, if a “protect the most vulnerable” strategy was applied, older patients with more aggressive cancer types and late-stage diagnoses whose treatment will only prolong their life expectancy for a limited time will be prioritized, albeit with potential consumption of the limited available medical resources, which may otherwise be directed to treat and save larger numbers of patients with better overall survival and better chances of benefiting from the treatment of their cancer in the long term. On the contrary, in the “save the greatest number of people” strategy, cancer patients with early stages, less aggressive cancer types, less complicated treatment regimens and higher chances of survival will be at the top of the list, which in the long run would result in more lives saved. For example, an old female patient with breast cancer with metastasis and co-morbidities would serve a good example for the third scenario i.e., protecting the most vulnerable. Whereas a young, otherwise healthy breast cancer patient with localized disease who stands a good chance of benefitting from an early treatment would fit into the first scenario; saving the most lives. Patients who have not yet been diagnosed or have been newly diagnosed and have not started treatment will be neglected in the “first come, first serve” strategy regardless of how life-saving the treatment can be to their case.

The United Kingdom's (UK) National Health Services (NHS) has issued its clinical guideline for the management of non-coronavirus patients requiring acute cancer care on 23rd March 2020. The guideline discussed different priority levels for categorizing patients undergoing surgery, patients on systemic anti-cancer treatments and patients on radiation therapy (Table 2) (8). It can be assumed that the NHS guidance has followed the “protect the most vulnerable” strategy for patients undergoing surgery as this would be judged based on the “emergency status” but a “save the greatest number of people” strategy for patients on systematic anti-cancer treatments as this would “result in the most lives saved” and a mix of both strategies for patients on radiation therapy.

**Table 2:** National Health Services (NHS) clinical guidelines for the management of non-coronavirus cancer patients (8).

**Priority level**	**Patient group**
**Patients undergoing surgery**	
Priority level 1a	Emergency—operation needed within 24 h to save life
Priority level 1b	Urgent—operation needed with 72 h Based on urgent/emergency surgery for life-threatening conditions such as obstruction, bleeding, and regional and/or localized infection permanent injury/clinical harm from the progression of conditions such as spinal cord compression
Priority level 2	Elective surgery with the expectation of cure, prioritized according to surgery within 4 weeks to save life/progression of disease beyond operability based on • urgency of symptoms • complications such as local compressive symptoms • biological priority (expected growth rate) of individual cancers
Priority level 3	Elective surgery can be delayed for 10–12 weeks will have no predicted negative outcome.
**Patients on systemic anti-cancer treatments**	
Priority level 1	• Curative therapy with a high (>50%) chance of success. • Adjuvant (or neo) therapy which adds at least 50% chance of cure to surgery or radiotherapy alone or treatment given at relapse
Priority level 2	• Curative therapy with an intermediate (20–50%) chance of success. • Adjuvant (or neo) therapy which adds a 20–50% chance of cure to surgery or radiotherapy alone or treatment given at relapse
Priority level 3	• Curative therapy of a low chance (10–20%) of success • Adjuvant (or neo) therapy which adds 10–20% chance of cure to surgery or radiotherapy alone or treatment given at relapse • Non-curative therapy with a high (>50%) chance of >1 year of life extension.
Priority level 4	• Curative therapy with a very low (0–10%) chance of success. • Adjuvant (or neo) therapy which adds a <10 chance of cure to surgery or radiotherapy alone or treatment given at relapse • Non-curative therapy with an intermediate (15–50%) chance of > 1-year life extension.
Priority level 5	• Non-curative therapy with a high (>50%) chance of palliation/temporary tumor control but <1-year life extension.
Priority level 6	• Non-curative therapy with an intermediate (15–50%) chance of palliation or temporary tumor control and <1-year life extension.
**Patients on radiation therapy**	
Priority level 1	• Patients with category 1 (rapidly proliferating) tumors currently being treated with radical (chemo)radiotherapy with curative intent where there is little or no scope for compensation of gaps. • Patients with category 1 tumors in whom combined External Beam Radiotherapy (EBRT) and subsequent brachytherapy is the management plan and the EBRT is already underway. • Patients with category 1 tumors who have not yet started and in whom clinical need determines that treatment should start in line with current cancer waiting times.
Priority level 2	• Urgent palliative radiotherapy in patients with malignant spinal cord compression who have a useful salvageable neurological function.
Priority level 3	• Radical radiotherapy for Category 2 (less aggressive) tumors where radiotherapy is the first definitive treatment. • Post-operative radiotherapy where there is known residual disease following surgery in tumors with aggressive biology.
Priority level 4	• Palliative radiotherapy where the alleviation of symptoms would reduce the burden on other healthcare services, such as hemoptysis.
Priority level 5	• Adjuvant radiotherapy where there has been complete resection of disease and there is a <20% risk of recurrence at 10 years, for example most ER positive breast cancer in patients receiving endocrine therapy. • Radical radiotherapy for prostate cancer in patients receiving neo-adjuvant hormone therapy.

The American Society of Clinical Oncology (ASCO) has created a series of Frequently Asked Questions (FAQs) to guide oncologists in their clinical practice during the SARS-CoV-2 pandemic (7). Other organizations have released guidance for specific cancer types, such as the American Society of Breast Surgeons (10, 11), the American Society of Hematology (12), and the Society of Surgical Oncology (13). Similarly, the European Society for Medical Oncology (ESMO) issued several guidelines on the management of various types of cancers, including for example, breast (14), lung (15), colorectal (16), and pancreatic carcinoma (17). Prioritizing cancer patients is based on a tiered framework that incorporated both the information on the value-based prioritization and clinical cogency of the interventions into a high, intermediate and low priority that would guide the surgical, medical, radiation interventions based on consensus recommendations from international experts.

Other parts of the world have made some efforts to develop recommendations to guide oncologists in providing cancer care during the SARS-CoV-2 in developing countries. Examples include collaborative work initiated through international collaboration, including contributions from some Arab Countries (18).

## Allocation of Limited Resources

The current pandemic has stretched healthcare resources in many ways. However, ventilators have stolen much of the show (19). If a SARS-CoV-2 infected cancer patient is competing with another SARS-CoV-2 infected, otherwise healthy, individual for a ventilator, how can one determine who gets the ventilator? A more complex situation can emerge for non-infected cancer patients who need the ventilator for their standard cancer care or terminally ill cancer patients who are already on ventilators; would such groups rank at the bottom of the list? In settings of scarcity such as these, it is important to consider not only what is ethically justifiable but also what is ethically unacceptable. Some may argue that removing terminally ill cancer patients already on ventilators to be used for SARS-CoV-2 infected patients with high chances of survival is ethically permissible, however, others may argue that it is ethically unacceptable especially without the consent of the patient or his/her family.

Since the emergence of SARS-CoV-2 pandemic, scientists are working day and night to find a potential treatment or vaccine to prevent the spread of the virus. Many anticipate the success of these treatments/vaccines to put an end to this tragic pandemic (19). However, this will not put an end to the currently faced ethical dilemmas. The significant question now will be who will have the priority to receive such treatments or vaccines? The dilemma of ventilators might propagate in case of establishing an effective treatment or an antiviral vaccine. For new vaccines, will priority be given to the most vulnerable to the infection/at higher risk of morbidity or mortality due to SARS-CoV-2 infection or to those who are most likely to benefit from immunization? In other words, will cancer patients be finally prioritized and seen as more vulnerable or will administer it to healthcare providers working in the frontline and interacting with hundreds of infected individuals on daily basis be more justifiable?

Whether it is a ventilator, antiviral medication, or vaccine, the consequences of a particular treatment decision can be afflictive for those excluded from benefit by that decision. Thus, no single person should be burdened to take such hard decisions. The value of well-educated, trained and experienced medical ethicists surfaces here. They are most-fit to balance such choices and guide the medical community to make the most justifiable ethical decisions governed by such specific circumstances. In addition, a clear ethical framework should be generalized and followed on a national level to ensure fairness of treatment. Fairness does not necessarily mean that every patient is provided with the same resources, rather differences in resource allocation, treatment and prioritization of patients is based on ethically justifiable criteria.

The American Medical Association (AMA) Code of Medical Ethics has provided foundation guidance in response to the SARS-CoV-2 pandemic (20). Table 3 lists the AMA Code of Medical Ethics Opinion 11.1.3, which gives guidance for allocating limited health care resources (21). It can be noticed that such guidance is framed broadly and intended to be applicable across a range of settings. A more specific framework is needed specifically to guide and unify the care provided for cancer patients during the current SARS-CoV-2 pandemic.

**Table 3:** American Medical Association (AMA) Code of Medical Ethics Opinion 11.1.3, Allocating Limited Health Care Resources (20).

Individually and collectively through the profession, physicians should advocate for policies and procedures that allocate scarce health care resources fairly among patients, in keeping with the following criteria:
(a) Base allocation policies on criteria relating to medical need, including the urgency of need, likelihood and anticipated duration of benefit, and change in the quality of life. In limited circumstances, it may be appropriate to take into consideration the amount of resources required for successful treatment. It is not appropriate to base allocation policies on social worth, perceived obstacles to treatment, patient contribution to illness, past use of resources, or other non-medical characteristics.
(b) Give first priority to those patients for whom treatment will avoid premature death or extremely poor outcomes, then to patients who will experience the greatest change in the quality of life, when there are very substantial differences among patients who need access to the scarce resource(s).
(c) Use an objective, flexible, transparent mechanism to determine which patients will receive the resource(s) when there are not substantial differences among patients who need access to the scarce resource(s).
(d) Explain the applicable allocation policies or procedures to patients who are denied access to the scarce resource(s) and to the public.

Acting and communicating ethically sound decisions should be a priority for healthcare providers during such hard times, and it becomes vital not only to communicate, but to provide resources of education for patients to help them make decisions regarding their treatment. However, during a crisis, the stakes grow higher, and the ethical challenges of communicating both accurately and strategically can be very complicated. Informed consents can be especially challenging. Additionally, in cases of scarce resources, physicians might need to play a proactive role and have premature end-of-life and DNR discussions with their cancer patients (6).

Another complex situation is when healthcare providers caring for cancer patients are called to care for critically-ill SARS-CoV-2 infected patients outside of their specialty and routine clinical practice, especially in a national health crisis (19). Here, physicians are left with a hard paradox of conscience leaving their own cancer patients, who they have been treating for years, juggling with their chances of survival after a long journey of painful procedures and treatment cycles, to fulfill yet another noble role and save many infected patients lives' giving them the opportunity to go back to their lives as healthy as they were before with no permanent negative consequences on their health.

## Situation in Jordan

On the 15th March 2020, Jordan had only one confirmed case of SARS-CoV-2 infection (22). Nonetheless, this did not stop healthcare institutions from starting to prepare for a potential health crisis, already witnessed in several countries worldwide at that time. No national guidelines for treating cancer patients were developed, leading individual institutions to take the initiative to develop their own internal policies.

### Situation at King Hussein Cancer Center

King Hussein Cancer Center (KHCC) is a standalone cancer center located in Jordan's capital, Amman. It provides comprehensive cancer care for the citizen of Jordan and neighboring countries. The center treats over 6,000 new cases annually; one quarter of which are non-Jordanians.

Given the unprecedented current outbreak and the lack of proper predictions on when such pandemic can be controlled, the diagnosis and treatment of malignant tumors should be carried out in an orderly and safe manner. Guidelines and recommendations on how to manage cancer patients during this pandemic do exist (18, 23). To meet challenges and to optimize quality care, KHCC had put into effect several measures:

#### Drive-Thru Screening

To avoid exposing our patients and our healthcare workers to SARS-CoV-2 infection, patients and their companions were screened twice; the day before their scheduled appointments to outpatient clinics, chemotherapy, radiotherapy, or elective diagnostic imaging, patients were screened over the phone by nurse coordinators about any exposure or clinical symptoms that may suggest SARS-CoV-2 infection. On the day of the appointment, all patients arriving at the center were screened again in a specially-designed “Drive-Thru” system where brief history and vital signs were measured.

#### Telemedicine

During the first 2 months of the pandemic, the center decided on adopting “Tele-Clinics.” All scheduled patients were notified the day before not to report to the hospital and that their clinic visits will be made via phone calls by their nurse coordinators and clinicians. During this “Tele-Clinic,” the team assessed patients clinically for all issues related to their cancer or its therapy. Occasionally, patients were requested to report to the hospital for a clinic visit, a drop-in clinic or even to the emergency room (ER). Such clinical encounters were documented in patients' electronic medical records.

Additionally, the center enforced and upgraded a previously established call center. Patients may call 24/7 inquiring about new complaints or issues related to their cancer or its therapy. Senior oncology nurses, who have access to all oncologists and other consultant physicians, operate this call center. Messages were also sent to all KHCC patients not to come to the ER before contacting the call center. Unnecessary ER visits were prohibited using this approach.

#### Limited Medical Services

During the first few weeks, KHCC limited elective surgeries and limited chemotherapy sessions to potentially curable cancers utilizing regimens not known to cause prolonged immunosuppression. Fortunately, these arrangements were temporary and resulted in minor delays in patients' active therapy. Likewise, the Hospital Ethics Committee updated and approved new modifications to the DNR policy to allow a team of physicians to make DNR decisions if more ICU beds or ventilators were needed (24). Fortunately, such situations were never encountered.

#### Medication Home Delivery

To avoid difficult commuting to the hospital and to minimize exposure, the center adopted a delivery plan to patients, to distribute newly prescribed and refilled medications. This service was welcomed by both patients and physicians alike. Special arrangements were made to refill narcotics as local rules and regulations prohibit delivering such medications.

#### Healthcare Workers

Learning from the experience encountered in some European and neighboring countries, the center decided to work during the early months with reduced staffing. Staff not on-duty were asked to stay home to avoid any accidental exposure and lengthy quarantines. An incidence of a single exposure at our center put aside more than 30 healthcare workers including physicians, nurses, dieticians, respiratory therapists, clinical pharmacists, housekeeping, and many others.

## Conclusions

As the current SARS-CoV-2 pandemic continues to evolve worldwide, many ethically challenging decisions must be made. This includes treating cancer patients, which might be easily overlooked at such difficult times. Much attention should be given to provide guidance for healthcare providers on delaying or altering cancer treatment plans, allocation of limited resources and patient-physician communication in addition to the importance of on-going discussions between medical ethicists and healthcare providers. The role of qualified medical ethicists and consultants is of paramount importance as they can ensure ethical medical practice during such critical times. However, in Jordan, such expertise are not abundant and the role of medical ethicists is still slowly emerging.

Finally, we provided a summary of the insight from the experience of KHCC, a comprehensive cancer center in this particular region. Overall, it appears that KHCC opted to adopt extreme measures to ensure the safety of patients and healthcare workers alike. As per the recommended triaging strategies (Table 1) it would appear that KHCC followed a mixture of “protecting the most vulnerable” and “prioritization of the most important.”

## Data Availability Statement

The original contributions presented in the study are included in the article/supplementary material, further inquiries can be directed to the corresponding author.

## Author Contributions

AA-T: literature review and collection of data, writing the first draft, review, and final approval. MA-H and AM: inception of the idea, critical review of the first draft, critical review, and final approval. RM and HS: literature review, reviewing, and editing the first draft, and final review and approval. HA-R: literature review, reviewing, editing the revised draft, and final review and approval. All authors are accountable for the content of the manuscript.

## Conflict of Interest

The authors declare that the research was conducted in the absence of any commercial or financial relationships that could be construed as a potential conflict of interest.
